# Prediction of Suicide Attempts Using the Box-Jenkins Model

**DOI:** 10.18502/ijps.v15i4.4296

**Published:** 2020-10

**Authors:** Yosra Azizpour, Kourosh Sayehmiri, Khairollah Asadollahi

**Affiliations:** 1 Department of Epidemiology, School of Health, Ilam University of Medical Sciences, Ilam, Iran.; 2 Department of Biostatistics, School of Health, Ilam University of Medical Sciences, Ilam, Iran.; 3 Department of Social Medicine, School of Medecine, Ilam University of Medical Sciences, Ilam, Iran.

**Keywords:** *Box-Jenkins Model*, *Epidemiology*, *Suicide Prediction*, *Ilam*

## Abstract

**Objective:** Suicide is a preventable social harm in the field of health. The present study aimed to design a prediction model for suicide incidence based on Box-Jenkins model in Ilam province.

**Method** **:** Using a retrospective method all completed and attempted suicide data were collected during 1993-2013. Then, using the autoregressive integrated moving average (ARIMA) model, the time series analysis of the Box-Jenkins model was conducted to predict suicide status in the coming years (2014-2015).

**Results: **According to the actual results obtained from the suicide data in 2014, a total of 1078 suicides were recorded and compared to the predicted results, according to the fitted model of the time series, which showed the selected model predicted 931 suicide cases, showing 86% accuracy of prediction. The series’ prediction indicated 931 suicides in 2014 with a negative growth rate of 25.3% compared to 2013 and 969 suicide cases in 2015 with a positive growth rate of 3.93% compared to 2014.

**Conclusion: **The results of this study showed the designed model provides a high diagnostic value to predict suicide rates. These types of models can help to predict suicide in future and plan to control and prevent suicide attempts.

Based on suicidal concepts, the incidence of suicide commitment is essentially to escape from an intolerable mental status (1). If a self-injury results in death, it is considered as a completed suicide; otherwise, it is only a suicide attempt (2). Suicide is a disastrous event with long-term and widespread effects on family, society, and even the entire country. A significant number of people are involved with suicide every year and having a history of suicide in general population is one of the important factors for suicide (3). According to the World Health Organization (WHO), there are about 800 000 completed suicides every year (3), and this figure is predicted to reach 1.5 million in 2020 (4). Heidari et al noted the risk of suicide was higher in Western Iran than parts of the country (5). Also, Kiadaliri et al reported the highest rate of completed suicide was found in Ilam, Kermanshah, Lorestan, and Hamedan provinces (West of Iran) (6). In the Americas and Europe (continents), the male to female ratio of completed suicide is 3.6 and 4, respectively (7). However, females have more suicide attempts than males in Iran, as a developing country (8). 

The time series is referred to the data collected on a variable in a regular, equal, and retrospective interval that helps to predict future events based on that data. In addition, time series analysis is the statistical method used for these data (9). Quantitative models are used to predict the time series and this is possible if the future prediction of event could be achieved based on the analysis of the time series components and their behaviors using mathematical methods (10). Different quantitative models have been introduced for analysis of time series; however, the Box-Jenkins model (ARIMA) is the most applicable tool for this purpose (11).

Bahadori Monfared et al used the ARIMA model to predict the mortality rate caused by traffic accidents in Iran and to provide a prevention model for traffic accidents. Their model showed about 93% accuracy (10). 

Other studies applying the ARIMA model to predict the incidence of Hand, foot, and mouth diseases (HFMD) (12), and hemorrhagic fever with renal syndrome in China (13) confirmed the high accuracy of disease forcasting via ARIMA model.

Given the due to the importance of suicide in the society, providing a statistical model for its prediction has a significant role in the prevention and reduction rate of this event. On the other hand, there is not a previous model to predict suicide in Ilam province. Hence, the present study was designed to predict total suicide rate, completed and attempted suicide during 2014 and 2015 using the Box-Jenkins model and then compare the predicted rate with the actual statistics in 2014.

## Materials and Methods

By a retrospective method, all monthly completed and attempted suicide data were collected during 1993-2013. All suicide information, including completed and attempted suicides data, is recorded in a suicide registration system in Ilam University of Medical Sciences (Farabar online program). Before the Farabar online system, the data were collected via paper forms and then the old information was entered into the new system. All relevant data from different medical centers are recorded monthly in Farabar and transferred to the main documentation center of the suicide committee. Then, the data are evaluated by the associated personnel and the repeated names are deleted before the final saving. Researchers use information according to the specific variables, including age, type of suicide, sex, time, etc. The patients' information is always kept confidential except for research or other purposes for which permission of authorities would be obtained. 

 A suicide patient may go directly to a hospital his/herself or is transferred by others. After a primary therapeutic approach, the reason for suicide and other relevant data is recorded based on the testimony of the patient or the individuals who accompanied her/ him, and the police are informed for any legal issues. Finally, the patient's data are entered into the Farabar system. If the patient is dead, Forensic examination is performed inside or outside the hospital to find the direct cause of death, after which a death certificate is signed. After Forensic specialist assessment, the method of suicide and the exact cause of death are added to the Farabar system by a nurse. If the deceased patients are referred to the legal medical centers directly, data are collected there via later communications and no case of completed suicide is missed, as a formal Forensic medical letter is mandatory for burial permission (14).

The time series analysis of the Box-Jenkins model was conducted using the ARIMA model to predict suicide status in the coming years (2014-2015). The model processing was divided into 2 stages of data: preprocessing and model evaluation.

***Data preprocessing***

The data preprocessing stage was as follows:

1. Outliers were identified using the t distribution with 95% confidence interval, and their values were replaced with a value obtained from linear coupling (15).
2. The time series plot was drawn using the STATA software.
3. The static nature of the data (eliminating the ascending and descending order of time series) is one of the primary conditions for data usage (16). Otherwise, nonstationary must be resolved (eliminating the trend from the time series as well as fixing the mean and variance). To eliminate nonstationary in the data, the differential was used once and the trend was removed from the data.
4. Appropriate models were fitted to the data.

***Evaluation of the model***

The statistical model evaluation was as follows:

1.   Determining the order of the model (p, q) is one of the other measures of the time series. The autocorrelation function (ACF) and partial autocorrelation function (PACF) plots were used (15).

2.   The ARMA(p, d, q) model was consider for prediction where ;

 p: The order of the autoregressive part 

          q:The order of the moving average part

          d: degree of differencing.

When independed variables are number of suicide in previous years and error, we must use time series models:

ARIMA prediction model is as follows:

Where;


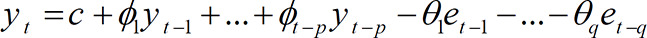


c = constant

Ø ᵢ = coefficient of each parameter P

Ø ᵢ = coefficient of each parameter q

e = residuals or errors in time t (17).

3.   In cases of using ARIMA models, the Akaikes’ information criterion (AIC) was used to select the superior model. According to this criterion, a model of the ARIMA family that had the minimum value was selected as the predictor (15).

4.   The time series prediction was performed using selected models for total suicide, suicide in females and males, and completed and attempted suicide for 2014 and 2015, and they were compared with actual statistics in 2014.

Note: Because we had 1 variable “number of suicide” in different years; thus, linear or nonlinear regression are not accurate enough to predict number of suicide in flowing years.

## Results

The best model for fitting the series was estimated to be ARIMA (0,1,18) for total suicide rate, ARIMA (0,1,11) for suicide rates in males, ARIMA (0,1,11) for suicide attempts in females, ARIMA (0,1,18) for attempted suicide rate, and ARIMA (0,1,18) for completed suicide rate (Table 1).

According to actual results obtained from the suicide data in 2014, a total of 1078 suicides were recorded and comparison of this rate with the predicted results, according to the fitted model to the time series, showed the selected model predicted 931 suicide cases, showing 86% accuracy of prediction. Also, 473 males’ suicide, 605 females’ suicides, 988 attempted suicides, and 62 completed suicides were recorded, and the prediction accuracy of the selected model for the mentioned variables was 83%, 87%, 88% and 97%, respectively (Table 2).

**Figure 1:**
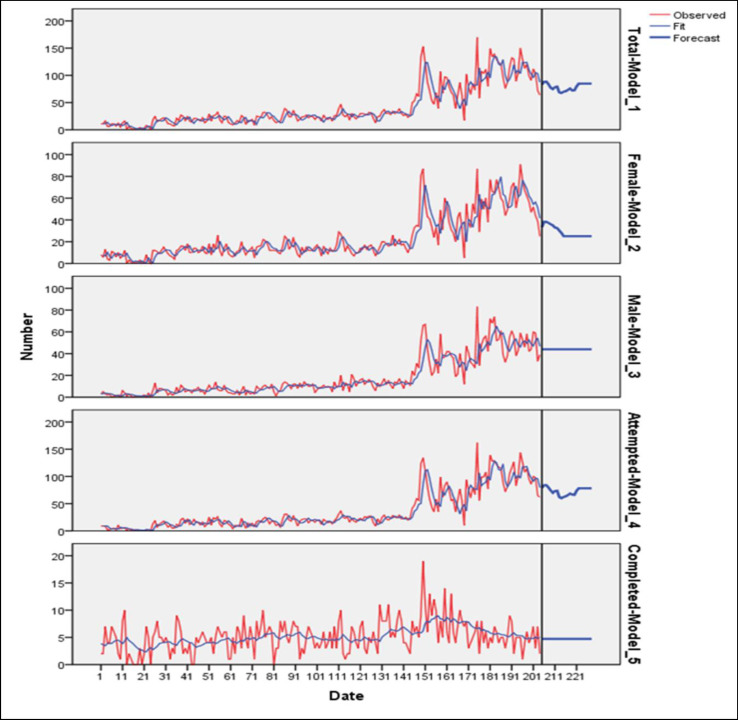
Predicted Rates for Total Suicide, Males’ Suicide, Females’ Suicide, Attempted Suicide, and Completed Suicide in 2014-2015

As it is shown in Figure 1, the series’ prediction indicated 931 suicides in 2014 with a negative growth rate of 25.3% compared to 2013. In 2014, there were 391 and 528 suicides in males and females with a negative growth rate of 32% and 21.5%, respectively, compared to 2013. Also, in 2014, there were 866 attempted suicides with a negative growth rate of 26.7% and 60 completed suicide attempts with a positive growth rate of 11.1% compared to 2013. For 2015, the series’ prediction indicated 969 suicide cases with a positive growth rate of 3.93% compared to 2014. In 2015, there were about 300 males’ suicides with a negative growth rate of 23.2% and 528 females' suicides with a growth of 0% (constant) compared to 2014. Also, in 2015, 888 attempted suicides occurred with a positive growth rate of 2.47% and 60 completed suicides with a growth of 0% (constant) compared to 2014.

**Table 1:** Characteristics of the Final Models Fitted to Attempted Suicide and Completed Suicide

**Variables**	**Models**	**Model Fit Statistics**	**Ljung–Box Q Test**		
		R-squared	Statistics	DF*	Sig
Total suicide	ARIMA* (0,1,18)	0.806	30.57	16	0.015
Males suicide	ARIMA (0,1,11)	0.756	30.67	16	0.015
Females suicide	ARIMA (0,1,11)	0.784	30.03	17	0.026
Attempted suicide	ARIMA (0,1,18)	0.819	30.08	16	0.018
Completed suicide	ARIMA (0,1,18)	0.112	10.57	17	0.878

* ARIMA: Autoregressive integrated moving average

**Table 2:** The Predicted Rates of Total Suicide, Males’ Suicide, Females’ Suicide, Attempted Suicide and Completed Suicide and Their Comparison with Actual Rates in 2014

**Variables**		**Total Suicide** **(0,1,18)** * ** ARIMA**	**Males’ Suicide** **ARIMA (0,1,11)**	**Females’ ** **Suicide** **ARIMA (0,1,11)**	**Attempted ** **Suicide** **ARIMA ** **(0,1,18)**	**Completed ** **Suicide** **ARIMA ** **(0,1,18)**
March	predict	83	33	44	79	5
	lower bound	50	13	27	48	0
	upper bound	116	53	61	110	10
	Real rate	122	57	65	111	5
April	predict	88	38	44	84	5
	lower bound	51	16	26	49	0
	upper bound	126	61	62	119	10
	Real rate	87	31	56	77	5
May	predict	89	38	44	84	5
	lower bound	48	14	25	46	0
	upper bound	129	63	63	122	10
	Real rate	106	52	54	96	7
June	predict	84	37	44	80	5
	lower bound	40	11	24	38	0
	upper bound	128	64	64	121	10
	Real rate	82	30	52	76	4
July	predict	77	36	44	75	5
	lower bound	30	8	23	30	0
	upper bound	124	64	65	119	10
	Real rate	118	52	66	109	5
August	predict	75	35	44	70	5
	lower bound	25	5	22	23	0
	upper bound	125	65	66	117	10
	Real rate	100	45	55	91	4
September	predict	78	33	44	73	5
	lower bound	25	1	21	24	0
	upper bound	131	65	67	123	10
	Real rate	88	39	49	83	4
October	predict	79	33	44	74	5
	lower bound	24	0	20	22	0
	upper bound	135	66	67	126	10
	Real rate	68	26	42	62	4
November	predict	69	30	44	62	5
	lower bound	11	0	20	8	0
	upper bound	127	65	68	116	10
	Real rate	77	31	46	69	8
December	predict	68	28	44	60	5
	lower bound	7	0	19	4	0
	upper bound	128	64	69	117	10
	Real rate	88	36	52	80	8
January	predict	70	25	44	62	5
	lower bound	7	0	18	4	0
	upper bound	132	63	70	121	10
	Real rate	74	42	32	69	5
February	predict	71	25	44	63	5
	lower bound	6	0	18	3	0
	upper bound	135	65	70	124	11
	Real rate	68	32	36	65	3

* ARIMA: Autoregressive integrated moving average

## Discussion

This study showed a decrease in the growth rate of total suicides, males and females’ suicide, and attempted suicide and a rise in completed suicide rates in 2014 compared to 2013. Also, the study indicated an increase in the incidence of total suicide and attempted suicide, a decline in males’ suicide rate and a constant rate of completed suicide and total females’ suicide in 2015 compared to 2014. There are various reasons for the increasing trend of suicide in Ilam province, which is not the subject of this article, but the factors such as increasing the class gaps, unemployment, poverty, and changes from the traditional to modern lifestyles may be among the reasons for the increase in suicides. In a province like Ilam, the lack of comprehensive systems to deal with tensions and the lack of the traditional social behaviors (communication with elders and relatives) to solve the suicide-related problems and deal with the psychological pressures that result from rapid socioeconomic change have led to an increase in suicides in this province (18). The total number of suicides in 2013 and in 2014 was 1252 and 1078, respectively, and the figure in 2014 was not increased and was even decreased to some extent, but their difference was not statistically significant. The annul suicide number per year is high for a sparsely populated province (population of 580 158 in the 2016 census). However, according to the results of the study, the accuracy of the models in predicting the desired variables is high. Hence, this statistical model can be used with a high confidence to predict suicide for the next years and could be applied in suicide prevention planning.

The components of a time series include trend, seasonal variations, periodic changes, and irregular changes. After estimating the trend, seasonal and periodic changes should be estimated as irregular changes if they occur based on unpredictable events (16, 19). The ARIMA model can have 2 nonseasonal and seasonal components, which is called seasonal ARIMA (SARIMA) and its equation is ARIMA (p, d, q) (P, D, Q) (20). However, in the present study, the time series followed an unstable random state and its equation was ARIMA (p, d, q). There was no seasonal trend in the data.

Pridemore and Snowden studied the reducing completed suicide after the implementation of the new alcohol policy in Slovenia between 1997 and 2005. The intervention model used to examine the effect of the studied policy on the total suicide was ARIMA (0.0,0) (0,1,1) 12), males’ suicide (ARIMA (0.0,0) (0,1,1)12, and females’ suicide (ARIMA (0,0,0). The researchers concluded after the implementation of the new policy on reducing alcohol consumption in 2003, there was a decreasing trend in both total suicide and male suicide rates between 2004 and 2006, but female suicide rates remained constant (21). Moreover, the study examined the impact of one factor on suicide, which may not be consistent with our study, but their study illustrates the strength of the ARIMA model to predict suicide, and we have used this model to predict suicide in the coming years.

## Limitation

There were some limitations in this study, including incomplete data on years 2000, 2002, 2007, and 2010 due to changing in the administrative team to collect data during these periods. Moreover, no valid data were available on unemployment rate and socioeconomic status during the study period in Ilam province to use them in the prediction models.

## Conclusion

In conclusion, the designed model, with the help of ARIMA model, was based on time series of total, males and females, attempted and completed suicide rates, and have a high potential for predicting suicide based on the mentioned variables for the future. However, applying suicidal prevention policies and using the prediction model for the impact of that policy in the coming years could be an interesting subject.
